# LMGAN: Linguistically Informed Semi-Supervised GAN with Multiple Generators

**DOI:** 10.3390/s22228761

**Published:** 2022-11-13

**Authors:** Whanhee Cho, Yongsuk Choi

**Affiliations:** Department of Computer Science, Hanyang University, Seoul 04763, Korea

**Keywords:** semi-supervised learning, semi-supervised GAN, text classification

## Abstract

Semi-supervised learning is one of the active research topics these days. There is a trial that solves semi-supervised text classification with a generative adversarial network (GAN). However, its generator has a limitation in producing fake data distributions that are similar to real data distributions. Since the real data distribution is frequently changing, the generator could not create adequate fake data. To overcome this problem, we present a novel approach for semi-supervised learning for text classification based on generative adversarial networks, Linguistically Informed SeMi-Supervised GAN with Multiple Generators, LMGAN. LMGAN uses trained bidirectional encoder representations from transformers (BERT) and the discriminator from GAN-BERT. In addition, LMGAN has multiple generators and utilizes the hidden layers of BERT. To reduce the discrepancy between the distribution of fake data and real data distribution, LMGAN uses fine-tuned BERT and the discriminator from GAN-BERT. However, since injecting fine-tuned BERT could induce incorrect fake data distribution, we utilize linguistically meaningful intermediate hidden layer outputs of BERT to enrich fake data distribution. Our model shows well-distributed fake data compared to the earlier GAN-based approach that failed to generate adequate high-quality fake data. Moreover, we can get better performances with extremely limited amounts of labeled data, up to 20.0%, compared to the baseline GAN-based model.

## 1. Introduction

A tremendous amount of data is necessary for the successful application of deep learning in the text domain [1]. Recently, enormous datasets are generally needed to obtain promising performances. Moreover, if provided with a large labeled dataset, powerful pre-trained large language models can offer great performances for specific tasks [2,3,4]. Yet, labeling data is a time-consuming and high-cost job. Thus, to overcome these problems, recent studies pay attention to semi-supervised learning for text classification. It tries to show comparable or better performance with few labeled text data and numerous unlabeled data compared to fully labeled text data.

Deploying data augmentation is one of the most popular methods for semi-supervised learning on text classification [5,6]. To augment the text datasets, state-of-the-art neural machine translator models and time-consuming translations are necessary for different datasets. Another method for semi-supervised learning on text classification is adversarial training [7] by applying a perturbation to input space [8,9]. Miyato et al. [8] injects perturbations to word embedding and Park et al. [9] requires input token replacements for injecting perturbations. Most state-of-the-art models change the input sentences.

Instead of these dominant lines of research on semi-supervised learning for text classification, this paper focuses on learning with generative adversarial networks (GANs). GAN-BERT pioneered applying GANs to a pre-trained language model for semi-supervised text classification [10]. GAN-BERT shows that a union of trained bidirectional encoder representations from transformers (BERT) and semi-supervised GANs can improve performance in semi-supervised text classification. Mostly, conventional GANs train with the fixed distribution of real data that a generator could make a similar distribution deceiving a discriminator. However, in the case of GAN-BERT, the real data does not have a fixed distribution. Since the real data are BERT [CLS] token output embeddings in the middle of fine-tuning, it has a frequently changing distribution while training.

**Figure 1:**
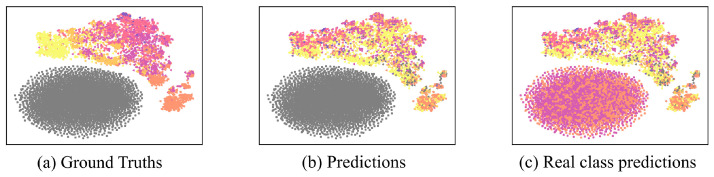
GAN-BERT data embeddings visualization with labels of ground truths (**a**) and predictions (**b**) represented by t-SNE. (**c**) shows results of classification without fake class. The colored version is better. They are the results of training the QC coarse-grained dataset with 1% labeled data. Gray color denotes the fake data class, and the other six colors mean six different classes, ‘ABBR’ (violet), ‘ENTY’ (magenta), ‘DESC’ (pink), ‘HUM’ (orange), ‘LOC’ (bright orange), and ‘NUM’ (yellow).

Figure 1 shows the embeddings of fake and real data with ground truths and predictions using t-SNE visualizations [11]. The images show 1% labeled GAN-BERT data embeddings in the QC coarse-grained dataset training with the same settings as [10]. Figure 1a,b represent ground truths and discriminator prediction results of real and fake data. Through the figure, the fake data embedding distribution is not similar to real data embedding distributions but only has round shape distributions. This indicates that the generator does not imitate real data distributions properly. Furthermore, we did not consider the k+1 class to show how the discriminator predicts an adequate label from {1,…,k} for the fake data in Figure 1c. The visualization results show that most fake data has mixed prediction results for a restricted number of classes. Hence, we can conclude that fake data does not create appropriate data embedding.

For these reasons, we assumed there could be a limitation for the generator to make good quality fake data. To address this limitation of the generator, we introduce Linguistically Informed SeMi-Supervised Generative Adversarial Networks with Multiple Generators, LMGAN. The proposed model enhances fake data quality with a hinted generator and hidden layers of BERT. The trained BERT from GAN-BERT conveys knowledge of candidate real data distribution that would delude the trained discriminator when a generator employs it. However, there is no guarantee that the trained BERT represents the correct sentence embedding distribution and the last layer of BERT has limited information about a sentence. Thus, LMGAN utilizes linguistically meaningful hidden layers of BERT with multiple generators for employing richer representations of fake data.

We evaluate LMGAN with various text classification datasets compared to baseline model performances. We analyze the impacts of selecting hidden layers of BERT to aid in imitating real data distribution. It reveals that some layer sets are beneficial to the performance. Moreover, we analyze the importance of each component of architecture and loss function. The analysis shows that deluding the discriminator with candidate BERT distributions causes the most improvements, meaning that the quality of fake data is significant comapared to the semi-supervised GAN.

Our contributions are summarized as follows:

- We introduce a novel approach for a semi-supervised GAN for text classification using a trained distribution of BERT to reach better distributions for fake data.
- We show improvements with multiple generators using linguistically meaningful intermediate hidden layers of BERT to a single generator using the last layer of trained BERT.
- LMGAN produces better fake data distributions than baseline. We analyzed the distributions by using t-SNE.

This paper first discusses a literature review of semi-supervised learning for text classification in Section 2.1 and semi-supervised generative adversarial networks to further explain how frequently trained embedding affects generative adversarial learning in Section 2.2. Moreover, this paper also describes related works on BERT’s hidden layer characteristics in Section 2.3. Section 3 proposes model architecture and methodology. Section 4.4 discusses the results of baselines and LMGAN. Furthermore, this paper goes on to validate the impact of the multi-generator and BERT’s hidden layers in Section 4.5.

## 2. Related Works

### 2.1. Semi-Supervised Learning for Text Classification

Recently, utilizing data augmentation outperforms semi-supervised text classification [5,6]. Unsupervised data augmentation (UDA) uses consistency regularization between unlabeled data and its augmentation results [5]. While training the model with unlabeled data, the model also trains with labeled data to maximize the probability of the correct class. UDA uses back translation for data augmentation, first from English text to French text and then from the result French text to English text. It derives the translations using WMT’14 English–French translation models. Another state-of-the-art model that uses data augmentation is MixText [6]. MixText handles unlabeled data with label guessing. The model classifies augmentations of unlabeled data and averages the possibilities over classes of augmentations. The class with the highest probability is the label of unlabeled data. Chen et al. [6] uses back translations for data augmentation, from English texts to Geman/Russian texts and from the translated texts to English texts. Intermediate outputs of BERT are mixed up whether the two data are labeled or unlabeled data. Then, the mixed data proceeds to the next BERT layer, and its output is used for cross-entropy loss. The labels are also mixed in the form of one-hot vectors with the same linear interpolation equation and hyperparameter.

Several studies suggest solving semi-supervised text classification with adversarial training [8,9]. Miyato et al. [8] first introduced adversarial training to semi-supervised text classification. The authors inject perturbation recurrent neural network word embedding. It helps the model robust from unseen examples and generalizes recurrent neural networks. Another method to apply perturbation to input space is token replacements [9]. The authors add perturbations, replacing tokens with results of augmentation methods to close semantic distances between adversarial examples and original sentences.

Unlike state-of-the-art models, GAN-BERT first adapts the semi-supervised generative adversarial network (SGAN) to semi-supervised learning for text classification [10]. Unlabeled data and labeled data are the input of BERT, and a discriminator utilizes output embeddings of BERT. Specifically, among outputs of BERT, the discriminator uses [CLS] token output embedding as real data from the semi-supervised GAN. Then, a fully connected layer generator makes fake data from random noise.

### 2.2. Semi-Supervised Generative Adversarial Networks

The SGAN learns how to classify unlabeled data with few labeled data [12,13]. The discriminator handles k+1 classes for semi-supervised learning. The discriminator classifies real data with *k* classes and discriminates real data and fake data with the k+1 class, which is a class of fake data. For the SGAN, real data are unlabeled data and labeled data. Unlike labeled data, which are annotated with *k* classes, unlabeled data are annotated with the k+1 class. Hence, the discriminator tries to predict it with any *k* classes.

The generator fools the discriminator with fake data with the same loss function as the original generator of the GAN, minimizing the probability that fake data are classified as fake data. In addition, SGAN uses feature matching [13]. The feature matching loss function could align statistics of the generator output and discriminator output. Feature matching is empirically proven to show great performance.

For semi-supervised GANs, two following conditions must be satisfied [14,15]:

(1) The discriminator has to be perfect at classifying labeled data.
(2) The generator can be imperfect at imitating but must be similar to the real data distribution.

GAN-BERT introduces using semi-supervised GANs on BERT [CLS] token output embeddings as applicable, showing performance improvements, and succeeds in the first condition of the above conditions. To meet the second condition, producing similar distribution to the real data distribution from the generator, we adopt the trained BERT [CLS] token output embedding from GAN-BERT to force the generator to know what the real data distribution is and the discriminator from GAN-BERT to injecting the knowledge that the discriminator thinks given fake data distribution is real.

Unlike traditional GAN, the distribution of real data of GAN-BERT is mobile. In other words, in training GAN-BERT, it fine-tunes BERT and uses the [CLS] token output of BERT, which frequently changes. Thus, while training GAN-BERT, the generator does not have genuine real data distribution as the amount of labeled data affects distributions.

Theoretically, a feature vector such as the [CLS] token output should represent sentence data with appropriate distribution. However, the amount of labeled data affects the ability of what feature vectors could illustrate. If the feature vector represents the data in the classification task, then the same class feature vectors on the feature space should be distributed closely. It means that the better quality of the feature vector in the classification task, the better the performance.

**Figure 2:**
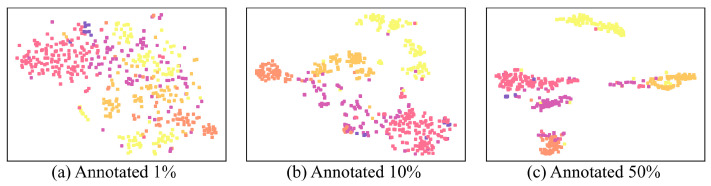
[CLS] token output distribution of the test dataset of the QC coarse-grained dataset. From (**a**) to (**c**), the model is trained with 1%, 10%, and 50% of data from the QC coarse-grained train dataset, respectively. The results show the distribution of the test dataset with six class colors, ‘ABBR’ (violet), ‘ENTY’ (magenta), ‘DESC’(pink), ‘HUM’ (orange), ‘LOC’ (bright orange), and ‘NUM’ (yellow).

Figure 2 shows t-SNE visualizations of BERT [CLS] token outputs with the different percentage of annotated data, 1%, 10%, and 50% of the QC coarse-grained dataset with 3 epochs and a learning rate of $2\times 10^{-5}$ Each case shows different distributions of data. Table 1 represents the performance of each experiment. The distribution of the [CLS] token output keeps changing since the [CLS] token output is just a feature vector. The [CLS] token output is a latent vector for the text classification, so it does not show real sentence distribution.

There are some trials that utilize latent space vectors while training GANs [16,17]. BiGAN uses real data as pair of the input image and input image embedding, and fake data as pair of fake image and fake image embedding [16]. GANomaly uses an autoencoder for the reconstruction of the real image as fake data [17]. While training GANomaly, the model minimizes differences between fake data and real data embedding. However, previous research eventually used the image as real data. Since GAN-BERT’s real data are latent representations of a sentence that is a [CLS] token embedding output, we cannot apply these prior works to GAN-BERT. Hence, we force the LMGAN generator to know the real data distribution from GAN-BERT to make the generator know the candidate’s real data distribution.

### 2.3. BERT Hidden Layers

BERT is famous for its astonishing performance in text data tasks [2]. However, besides receiving attention from the fact that it outperforms explainable artificial intelligence (XAI) [18], analysis of BERT’s hidden layers is also studied. Recent researches find that each BERT layer can learn different information from sentences [6,19,20,21].

For example, layer 12 is sensitive to knowing the replacements of nouns and verbs, layer 9 learns about the subject and verb relations over long distances, layer 7 comprehends top-level sequences in a syntax tree, and layer 6 understands the depths of syntax trees [19]. Furthermore, layer 11 and layer 12 learn remarkably different information from the original BERT while fine-tuning. This indicates that these layers discover task-specific information. On the contrary, lower layers have similar weights to the original model. Thus, they are learning about basic linguistic structure information [20].

Even if LMGAN injects the distribution of the trained BERT into the generator and fools the trained discriminator, there are two problems because of the information from the trained BERT. First, there is a possibility that the incorrect distribution of the trained BERT could interrupt the generator to create adequate fake data. The problem happens because the BERT from GAN-BERT knows incomplete sentence distribution since it is trained with limited annotated data. Moreover, the BERT last layer output shows restricted linguistic characteristics. When utilizing sentence representation from BERT, conventionally we only use the final layer [CLS] token output vector, which contains limited linguistic information compared to the other layers [19,20,21].

Hence, like augmentation, we use intermediate hidden layers of BERT to express the same sentence with different representations to make richer fake data distributions with various linguistic information. To employ hidden layer [CLS] token embeddings, we use multiple generators. By using various generators with different hidden layer outputs, we could expect the likelihood that one of the fake data distributions becomes similar to the real data distribution would be higher than using only one generator. Furthermore, the discriminator and BERT could find better classification distributions since they look at linguistically various fake data distributions. However, if the information in BERT’s hidden layer is not helpful, it could interrupt learning. Hence, in Section 4.4 we validate that linguistically meaningful sentence representations help classification performance.

## 3. Methods

**Figure 3:**
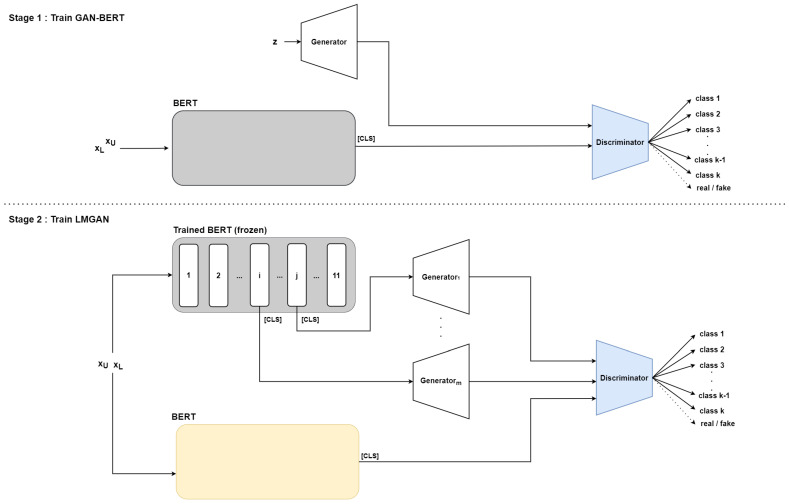
LMGAN architecture and training methods. The colored version is better. Training steps consist of two steps. A trained BERT and discriminator at stage 1 are utilized for fake data for LMGAN at stage 2. $x_{L}$ means labeled data and $x_{U}$ means unlabeled data. At stage 2, the same BERT and discriminator parameters are used as at stage 1, thus using the same colors for the modules.

We introduce our novel method of semi-supervised approach for text classification, Linguistically Informed Semi-Supervised GAN with Multiple Generators, LMGAN. Figure 3 describes the overall architecture of our model. As shown in the figure, our model has two stages. In the first stage, stage 1 in the figure, we first fine-tune BERT and train the discriminator using GAN-BERT. This step is necessary for the inputs of generators. In the second stage, stage 2 in the figure, the fine-tuned BERT is used for generator input. Additionally, we use a trained discriminator from GAN-BERT to alter the output of the trained BERT. The discriminator discriminates whether the data are fake or real and classifies labeled data while fine-tuning another BERT. In addition, there are multiple generator inputs for utilizing linguistic information. We use the discriminator and the second BERT when evaluating the model.

As mentioned in Section 2.3, BERT’s hidden layers can contain various linguistic information [6,19,20]. Hence, applying this information to generators would yield distinct fake data distributions that can increase the probability of making distributions similar to real data distributions.

To utilize the linguistic information of the dataset from BBERT’s hidden layers, we first train BERT with GAN-BERT, as shown in stage one of Figure 3. Training BERT from GAN-BERT sentence embeddings will provide more information about the dataset instead of using embeddings from the vanilla pre-trained BERT. Since prior research discovered useful results after fine-tuning BERT [19,20], we fine-tune BERT with a discriminator that classifies labeled data with classes *k* and discriminates real and fake data. As Jawahar et al. [19] uses [CLS] token embedding for learning and Kovaleva et al. [20] shows [CLS] token is one of the important tokens among other tokens, we use [CLS] token embedding for classification.

Then in stage 2, we use fine-tuned BERT at the generators. Some outputs of BERT’s 12 layers are selected as inputs of the generators, as shown in Figure 3. Thus, there are equivalent numbers of selected BERT layers and generators. Each selected hidden layer output [CLS] token embedding goes to different generators. It can be formulated as Equation (1):

(1) $${\tilde{x}}^{i}=Pool\left(E^{i}\left(x\right)\right),i\in \left[1,12\right]$$

In Equation (4), $E^{i}\left(x\right)$ is the *i*th BERT hidden layer output and Pool is a pooling function that pools [CLS] token embedding from the hidden layer. The result, ${\tilde{x}}^{i}$, becomes the input of a generator that conveys different lingual data information from other generators with the same sentence. Thus, there are *m* different representations from generators for one sentence with an equal class.

### Semi-Supervised Learning

As Croce et al. [10] proposes, we use a semi-supervised GAN for semi-supervised text classification. In addition, LMGAN uses two additional objective functions for training generators: the cross-entropy function for labeled data, and the KL divergence function for unlabeled data. First, the discriminator discriminates whether the input is real or fake. On the other hand, the generator of the GAN fools the discriminator, making the fake data more realistic [22]. Equations (2) and (3) show loss of generator and discriminator when *m* is the model, and when $\tilde{x}$ is fake data from the trained BERT and *x* is real data from vanilla BERT.

(2) $$\begin{array}{cc} L_{D} & =-E_{x∼p_{x}}\left[log\left(p_{m}\left(x\right)\right)\right]-E_{z∼p_{z}}\left[1-log\left(p_{m}\left(z\right)\right)\right] \end{array}$$

(3) $$\begin{array}{cc} L_{G} & =-E_{x∼p_{\tilde{x}}}\left[log\left(p_{m}\left(x\right)\right)\right] \end{array}$$

Moreover, the semi-supervised GAN learns how to classify unlabeled data with a small amount of labeled data with GAN-based objective functions [12,13]. A discriminator handles k+1 classes for semi-supervised learning. The discriminator classifies real data with *k* classes and discriminates real data and fake data with k+1 class, which is a class of fake data. For the SGAN, real data are unlabeled data and labeled data. Unlike labeled data, which are annotated with *k* classes, unlabeled data are annotated with k+1 class. Hence, the discriminator tries to predict it with any *k* classes.

Thus, the discriminator loss function includes two parts, supervised learning loss and unsupervised learning loss function, as shown in Equation (4). Supervised learning, $L_{sup}$, classifies labeled data with *k* classes, as in Equation (5) where *m* is a model, and unsupervised learning $L_{unsup}$ discriminates fake data and real data, as shown in Equation (6). In $L_{unsup}$, real data consist of labeled and unlabeled data.

(4) $$\begin{array}{cc} L_{D}= & L_{sup}+L_{unsup} \end{array}$$

(5) $$\begin{array}{cc} L_{sup}= & -E_{x∼p_{x_{data}}}\left[log\left(p_{m}\left(x\right)=k\right)\right] \end{array}$$

(6) $$\begin{array}{cc} L_{unsup}= & -E_{x∼p_{x}}\left[log\left(p_{m}\left(x\right)<k+1\right)\right]-E_{x∼p_{\tilde{x}}}\left[log\left(p_{x}\left(x\right)=k+1\right)\right] \end{array}$$

The generator fools the discriminator with fake data with the same loss function as the original generator of the GAN, as in Equation (9). The generator tries to make similar distributions of real data, minimizing the probability that the discriminator distinguishes the fake data as fake data. In addition, SGANs use feature matching [13], formulated as Equation (8), where f is the discriminator. By adding feature matching to generator loss, the distribution of the generator output and the discriminator could be aligned. Feature matching is empirically proven to show great performance [13]. Hence, the loss function of the generator can be defined as Equation (7).

(7) $$\begin{array}{cc} L_{G}= & L_{fm}+L_{unsup} \end{array}$$

(8) $$\begin{array}{cc} L_{fm}= & \left|\right|E_{x∼p_{x_{data}}}f\left(x_{data}\right)-E_{x∼p_{\tilde{x}}}f\left(x\right){\left|\right|}^{2} \end{array}$$

(9) $$\begin{array}{cc} L_{unsup}= & -E_{x∼p_{\tilde{x}}}\left[1-log\left(p_{m}\left(x\right)\right)\right] \end{array}$$

Since LMGAN has multiple generators, we need to modify the generator loss function. Each generator receives the same sentence but different linguistic representations. However, they have the same label. We assume that each generator output has various manifold embeddings from the same sentence with the same class. To make each generator output have the same label, we averaged the generator distribution outputs of the discriminator as earlier research averaged augmentation data distribution for guessing label [6,23]. This can be formulated as Equation (10), where $x^{i}$ is the trained BERT *i*th layer output.

(10) $$p_{avg}\left(x\right)=E_{x∼p_{\tilde{x}}}\left[D\left(G_{1}\left(x^{1}\right)\right),\ldots ,D\left(G_{m}\left(x^{m}\right)\right)\right]$$

When the input sentence of the generator is labeled data, each generator maximizes ground truth class probability as Equation (11). On the contrary, when the input sentence is unlabeled data, we do not know the class of the data. Hence, LMGAN guesses the correct label with pseudo-labeling [6]. With guessed labeling, the generator minimizes Equation (12).

(11) $$\begin{array}{cc} L_{sup}= & -E_{x∼p_{\tilde{x}}}\left[\log p_{avg}\left(x\right)=k\right] \end{array}$$

(12) $$\begin{array}{cc} L_{KL}= & -KL(p_{avg}\left(x\right)||D\left(G_{i}\left(x^{i}\right)\right),i\in \left[1,m\right] \end{array}$$

We combine divergence loss, supervised learning loss, and Equation (8) to train the generator, as in Equation (13):

(13) $$L_{LMGAN_{G}}=L_{fm}+L_{unsup}+L_{KL}+L_{sup}$$

The LMGAN discriminator loss is the same loss function as the SGAN discriminator. When the generator outputs are going to be inputs of the discriminator-like batch, all fake data are the input. It is formulated as Equation (14):

(14) $$L_{LMGAN_{D}}=L_{D}$$

Hence, the LMGAN loss function is the sum of the generator loss and discriminator loss:

(15) $$L_{LMGAN}=L_{LMGAN_{D}}+L_{LMGAN_{G}}$$

## 4. Results and Discussion

### 4.1. Datasets

We evaluated our model on various text classification datasets. We estimate LMGAN with the same set and settings of datasets from GAN-BERT [10]. We use the QC coarse-grained and QC fine-grained datasets for question classification task [24], the 20 News Group dataset for topic classification [25], the SST-5 dataset for sentiment classification [26], and the MNLI dataset for natural language inference [27]. We used accuracy for the QC coarse-grained, QC fine-grained, and SST-5 datasets and F1 score for the 20 News Group and MNLI datasets [10].

The QC coarse-grained and QC fine-grained datasets are for question classification and have the same data. The only difference between them is the number of classes. The QC coarse-grained dataset has six classes, and the fine-grained dataset has 50 classes. The amount of train and test data are based on the baseline setting [10]. The QC dataset has 5500 train data and 500 test data. The 20 News Group dataset is for topic classification with 20 classes, 11,314 train samples, and 7531 test samples. SST-5 is for sentiment classification with 5 classes, 8544 train samples, and 2210 test samples. MNLI is for inference classification with 3 classes, 392,702 train samples, and 10,000 test samples. The MNLI dataset could be evaluated with two different development sets, matched samples and mismatched samples. Matched samples are derived from the same sources as training samples and mismatched samples are not. The summary of the statistics of the datasets is described in Table 2.

We conduct experiments with different amounts of labeled data and unlabeled data, changing the annotated data percentage of a dataset as in the settings in [10]. For QC, SST-5, and 20 News Group, we changed annotated data to 1%, 2%, 5%, 10%, 20%, 30%, 40%, and 50% of the dataset. In the case of the MNLI dataset, we changed annotated data to 0.01%, 0.02%, 0.05%, 0.1%, 0.2%, 0.5%, and 1% of the dataset. Such annotated data are trained with Equation (5) and the rest of the unlabeled data are trained with Equation (6).

### 4.2. Baseline Models

To validate the performances of LMGAN, we compared performances with two models: BERT and GAN-BERT.

Bidirectional encoder representations from transformers (BERT) [2] is a language model that comprises only self-attention encoders from a transformer [28]. This model is pre-trained in two ways, masked token prediction with a randomly masked token and next sentence prediction with a [CLS] token. BERT output represents the context of a sentence, mainly with [CLS] token output. We use a pre-trained BERT-based uncased model that is pre-trained with lowercase English language and 12 self-attention layers. We fine-tuned BERT with a small amount of labeled data using [CLS] token embeddings.

GAN-BERT [10] is for the label classification of unlabeled data and labeled data using a semi-supervised GAN [13] and BERT [2]. GAN-BERT first utilizes BERT [CLS] output of data for sentence vectorization. After BERT changes a sentence to representation, the discriminator predicts labels of labeled data BERT [CLS] output and classifies whether input data are fake or real. The generator makes fake data from random noise and tries to fool the discriminator. We experimented GAN-BERT model from the original paper code. We experimented with the GAN-BERT model from the original paper’s code.

### 4.3. Detailed Settings

Except for the framework of generators, our model uses the same architecture as GAN-BERT. We used a BERT-based uncased tokenizer for tokenizing our text data and a BERT-based uncased model for the pre-trained BERT of huggingface, respectively. We used [CLS] token embedding from the output of BERT for GAN-BERT and LMGAN. LMGAN uses one fully connected layer for each generator with 768 dimensions and a LeakyReLU activation function. For all outputs of the fully connected layer, we used dropout with a rate of 0.1. LMGAN uses two fully connected layers for the discriminator. One layer receives data from the generator and real data outputting features with dimension 768. The other layer obtains the features and returns the number of classes, which is k+1. Additionally, we trained BERT with the huggingface BertForSequenceClassification model. The epochs and maximum length are the same as the settings in [10].

When learning LMGAN, in phase one, we fine-tuned BERT and trained the discriminator with a learning rate of $2\times 10^{-5}$ for all datasets with three epochs, except 20 News Group. We trained GAN-BERT with a learning rate of $5\times 10^{-6}$ and 15 epochs [10] with the same data settings. In the second phase, we used the trained BERT from GAN-BERT as an inference at the generator input. However, the non-fine-tuned BERT is used for the real data. These two BERTs do not have the same parameter values. We selected the sixth, seventh, and ninth hidden layers of BERT for the generators since the combination showed great performance.

We evaluate LMGAN with the same learning rate and training epochs as GAN-BERT [10], except for 20 News Group. The learning rate for the discriminator and the generator is $5\times 10^{-6}$ for 20 News Group, and $2\times 10^{-5}$ for the QC coarse-grained, QC fine-grained, SST-5, and MNLI datasets. We used AdamW optimizer from huggingface transformers. We trained each dataset with three epochs.

Croce et al. [10] duplicated labeled data to guarantee that each min-batch carries at least one labeled datum for stable training. However, duplicating labeled data does not assure every batch includes labeled data. Thus, we set each batch to have one labeled datum if there are limited annotated data. Furthermore, if there are lots of annotated data, we compose batches to have labeled data and unlabeled data evenly as most semi-supervised learning handle batches in this way [5,6,13]. We trained GAN-BERT and LMGAN with this strategy.

### 4.4. Results

**Figure 4:**
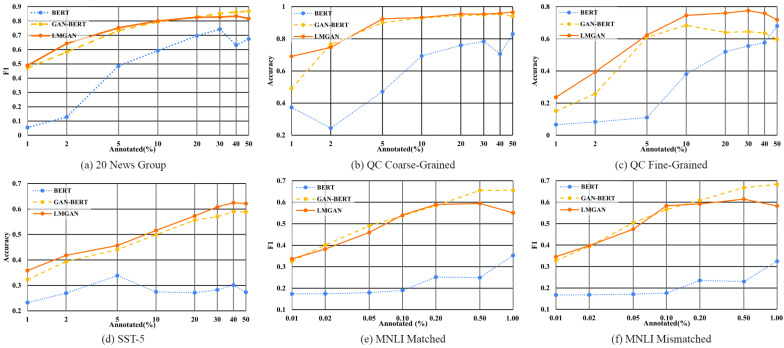
Performance plots of baselines and LMGAN with four tasks, changing the amount of annotated data.

We apply our model to various text datasets with different amounts of annotated data. In phase one, we trained the baseline with the same data settings and used its trained BERT and discriminator for LMGAN. Figure 4 shows performances of BERT, GAN-BERT, and LMGAN. In addition, we append loss plots of GAN loss functions (4) and (7) for GAN-BERT and LMGAN in Appendix A. In most results of datasets, our model outperformed GAN-BERT. Our model shows great improvements with extremely low amounts of labeled data. When there are the least annotated data, LMGAN improved by 1.7% for 20 News Group, 20.0% for QC coarse-grained, 8.6% for QC fine-grained, 3.6% for SST-5, 1.2% for MNLI matched, and 1.7% for MNLI mismatched comapred to GAN-BERT. Moreover, except the MNLI dataset, when there are lots of labeled data, LMGAN shows comparable or better performances than GAN-BERT. In addition, we could observe divergent performances in almost every BERT performance when the amount of labeled data increases. We can assume that BERT is sensitive to the amount of labeled data.

The results of 20 News Group show that LMGAN is more powerful than GAN-BERT at semi-supervised learning for topic classification when there are few labeled data. Our model achieves 48.9%, 64.3%, and 75.3% when there are 1%, 2%, and 5% annotated data (about 110, 220, and 550 labeled examples) and GAN-BERT reports 47.2%, 58.3%, and 73.2% F1 scores. Moreover, LMGAN shows many improvements over BERT. When there are 1%, 2%, and 5% annotated data, LMGAN showed 12.6%, 14.8%, and 11.8% improvements. However, after 10% annotated data, LMGAN converges, unlike GAN-BERT, and shows slightly worse performances. We believe this happens since the number of epochs of learning for 20 News Group is bigger than the rest of the datasets, and the trained BERT and discriminator would be overfitted.

The performance of QC coarse-grained is shown in Figure 4b. LMGAN shows 69.1% and 92.4% accuracy, whereas GAN-BERT is 49.3% and 90.0% when there are 1% and 5% labeled data (about 50 and 250 labeled examples). At these data settings, our model achieves 20.0% and 2.3% improvements. Except for 2% annotated data, LMGAN shows better performances than GAN-BERT. Furthermore, LMGAN shows great improvements from BERT. When there are 1%, 2%, and 5% annotated data, 31.7%, 50.3%, and 45.2% performances are achieved.

LMGAN’s performance on QC fine-grained shows great improvements whenever there is a large amount of annotated data. LMGAN shows 23.6% and 39.4%, whereas GAN-BERT is 15.1% and 25.8% when there are 1% and 2% labeled data (about 50 and 250 labeled examples). Moreover, when there are 40% and 50% annotated data, LMGAN shows more than 10% accuracy improvements over GAN-BERT. In addition, our model shows great performances compared to BERT where when there are 1%, 2%, and 5% annotated data: our model showed 17.0%, 31.1%, and 51.4% improvements.

On sentiment classification, the SST-5 dataset, we found that LMGAN results in accuracy improvements over most annotated data. LMGAN shows accuracies of 35.9% and 41.8%, whereas GAN-BERT’s accuracy is 32.3% and 39.3% when there are 1% and 2% annotated data (about 85, and 170 labeled examples). However, BERT shows significantly worse performance on limited amounts of annotated data. It shows that BERT needs large amounts of labeled data to be properly trained.

On natural language inference, for the MNLI dataset, we observed that GAN-BERT’s performance is mostly better than LMGAN. We assume that LMGAN is focused on making semantically or syntactically rich sentence representations and understanding a single sentence for classification since we followed the results of hidden layers of a single sentence. However, the purpose of text classification on MNLI is to know the relationship between two sentences; the hypothesis is that there will be no need to offer fake linguistically different sentence representations that are only meaningful in a single sentence.

### 4.5. Linguistic Information of BERT Hidden Layers

To prove the claim that BERT’s hidden layer linguistic information could help limited information from the trained BERT, we built different sets for generator input to show the impact. Since meaningless intermediate hidden layer sentence representations could interrupt adversarial learning, we assumed that utilizing adequate linguistic information of BERT’s hidden layers would increase the possibility of fake data similar to real data completing lacking fake data distribution and sentence representation. By improving the generation of fake data distribution or quality, we could overcome the second condition of training the SGAN. Thus, if hidden layer outputs show various sentence representations, the classification performances would be increased. To prove this, we created sets with three categories based on performance over probing tasks [19]: layers with poor performances, first-ranked layers, and high-ranked layers over the dataset. As mentioned, we assumed hidden layer outputs showing appropriate meaningful sentence representation would provide great performances, such as high-ranked layer sets. Moreover, we compared the performance of sets to BERT’s last layer performance, set {12}.

Table 3 describes the LMGAN accuracy of 1% labeled data for QC coarse-grained with the different layer sets for generators. We selected layers from BERT’s 12 layers using the results from [19]. Set {1, 2}, {1, 2, 3}, and {1, 2, 3, 4} is the poor performance layer set, set {9, 12}, {6, 9, 12}, and {6, 8, 9, 12} is first-ranked layer set, and set {6,9} and {6, 7, 9} is the high-ranked layer set. In the case of the QC coarse-grained dataset, we observed that the layer with set {6, 7, 9} shows the best performances compared to set {12}, as we expected. For every different amount of annotated data, except at 30% annotated, the set shows better performances than set {12}. In the case of a poor performance layer set, we could observe that when there are few annotated layers, the performance was worse than set {12} and when there are large amounts of labeled data, set {1, 2, 3} shows the best performances than other sets at 20%, 40%, and 50% annotated data. In the case of the first-ranked layers set, except set {6, 8, 9, 12}, we could see that the performances are worse than the performances of set {12}. However, most of the sets with large amounts of labeled data showed comparable performances to set {12}.

In the case of the SST-5 dataset in Table 4, the layer set showed a similar tendency. However, we could observe that the impact of layers is weaker than the QC coarse-grained set. Moreover, we discovered that set {1, 2, 3} showed the best performances along with set {6, 7, 9}. We believe that even if the layers of set {1, 2, 3} showed poor performances on probing tasks and set {1, 2} and set {1, 2, 3, 4} showed inferior performances in the table, the unique combination of {1, 2, 3} could mean something linguistically valuable.

Hence, employing intermediate hidden layers could show better performances depending on the amount of labeled data and the selection of layers. Layers that are good at overall probing tasks showed the greatest performances. Hence, we can conclude that adequate sentence representation could improve classification performance. Moreover, we propose improvements with multiple generators using the intermediate hidden layers of BERT to use the last layer of the trained BERT.

**Figure 5:**
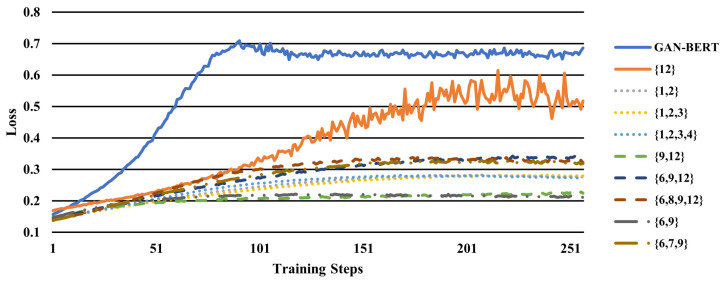
Generator unsupervised loss plots of GAN-BERT and LMGAN with various intermediate hidden layer sets. It is the loss of training 1% annotated data on QC coarse-grained.

As well as observing the performances to check whether the intermediate hidden layers are useful for the model, we plotted unsupervised loss of generators to inspect whether fake data are fooling the discriminator, using Equation (9). If the loss is small, it implies that the fake data are deceiving the discriminator well. Thus, this could be evidence that the fake data are generating similar distribution to the real distribution. Figure 5 describes the generator loss of generators with different hidden layer combinations from Table 3 and Table 4 and GAN-BERT. Compared to the GAN-BERT generator, all the set combinations fool the discriminator better. In addition, we can observe that diverse fake data fools the discriminator more than just using the last layer output. However, for example, even if the data are fooling discriminator well as in set {1, 2, 3, 4} or {9, 12}, as shown in Table 3 and Table 4, the performance could be bad. Thus, we can assume that the quality of linguistic information is important for fake data.

### 4.6. Ablation Study

We performed ablation studies by removing each part of LMGAN to validate our contributions. Through ablation analysis, we mainly show that utilizing the trained BERT and the trained discriminator from GAN-BERT improves performance. Moreover, the loss functions help the generators to learn real data distribution. The experiments are on QC coarse-grained with 1% annotated data with three different seeds, as shown in Table 5. We averaged the outputs of three random seeds. We removed the trained BERT, trained discriminator, both trained BERT and the discriminator of GAN-BERT, and multiple generators. Furthermore, we eliminated cross-entropy loss (11), KL divergence (12), and both loss functions at the generators to show the impact of our model.

We assumed the baseline generator has a limitation for making high-quality fake data because of the frequently changing real data distribution, we added trained BERT and trained discriminator of GAN-BERT for the generator to make the fake data easier. To validate the assumption, we removed both of them. Then, only the pre-trained BERT would be the input of generators. In this case, real data distribution starts to train with the same parameter with generator input. Hence, the generator makes fake data from a fixed BERT distribution, except it is a possibly correct classification distribution. As it has a fixed distribution of BERT, the model trains a semi-supervised GAN more powerfully than our baseline. Additionally, removing multiple generators shows lower performance than LMGAN since multiple generators help the real data BERT to make a better classification distribution.

When removing the trained BERT, only the trained discriminator is left, which still thinks of the real data distribution as earlier learned distribution. Thus, removing the trained BERT still improves the performance. However, removing the trained discriminator of GAN-BERT results in the most differences. The trained discriminator helps adversarial learning much more effectively since the vanilla discriminator does not think the fake data are real. Hence, using a fixed distribution of BERT and letting the discriminator think the fake data distribution is real helps the adversarial learning of the semi-supervised GAN.

We observe that removing loss functions shows differences, and each loss is influential. Excluding cross-entropy loss showed much more difference than kl divergence loss. We believe this is because guessing correct labels is much easier than guessing labels with KL divergence. Moreover, eliminating both loss functions harm the classification performances the most.

### 4.7. Embedding Visualization

**Figure 6:**
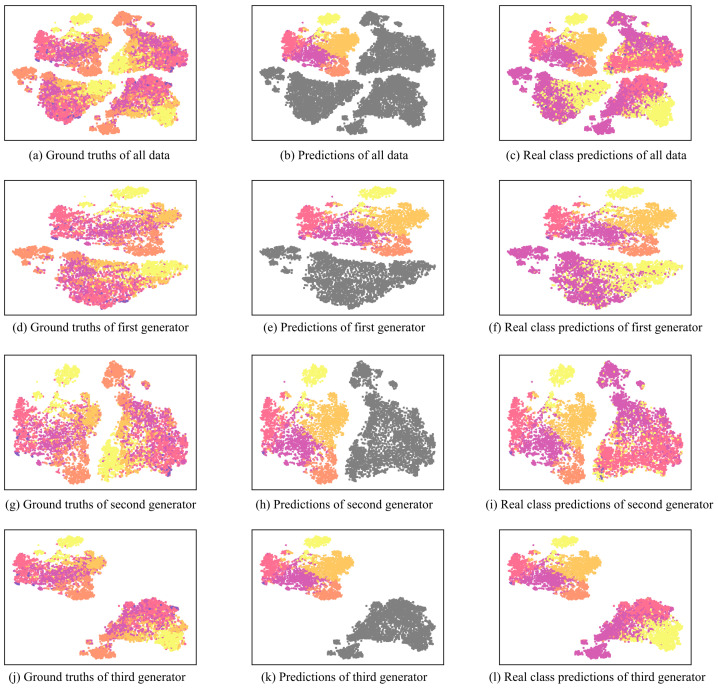
LMGAN with {6,7,9} layer data embeddings with labels of ground truths and predictions represented by t-SNE. The colored version is better. The image shows LMGAN data embeddings with ground truths and class predictions of the QC coarse-grained dataset with 1% labeled data. From left to right, the images show ground truths and predictions of fake and real data. Gray color denotes fake data class and the other six colors mean six different classes, ‘ABBR’ (violet), ‘ENTY’ (magenta), ‘DESC’ (pink), ‘HUM’ (orange), ‘LOC’ (bright orange), and ‘NUM’ (yellow). From top to bottom, the image shows the outputs of three generators and each generator.

To show how LMGAN’s fake data distribution is shaped, we used t-SNE for visualizing the data. Figure 6 is the LMGAN data embeddings of fake data and real data represented by t-SNE on 1% labeled data of the QC coarse-grained dataset with three generators. There are seven colors in the figure. The gray color means the fake data class, and the others are real data class. In the figure, from left to right, they are ground truths of the data, prediction results of the discriminator, and prediction results without the fake data class. In this experiment, we used the {6,8,9} layers of BERT and three generators. The first row is about all the fake data from the three generators, and the other rows show the first, second, and third generator’s fake data embeddings. Each image shows the results of ground truths of one generator utilizing the sixth hidden layer [CLS] token embedding of BERT and the [CLS] token embedding of BERT. The second row shows the result of the predictions of the discriminator.

**Figure 7:**
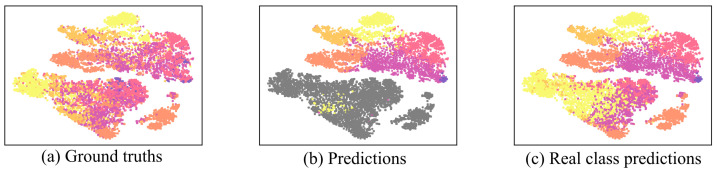
LMGAN with {12} layer data embeddings with labels of ground truths and predictions represented by t-SNE. The colored version is better. The image shows LMGAN data embeddings only using the last layer of the trained BERT. Performances of the QC coarse-grained dataset with 1% labeled data. From left to right, images show ground truths and predictions of fake and real data. Gray color denotes fake data class and the other six colors mean six different classes, ‘ABBR’ (violet), ‘ENTY’ (magenta), ‘DESC’ (pink), ‘HUM’ (orange), ‘LOC’ (bright orange), and ‘NUM’ (yellow).

As shown in Figure 6, LMGAN shows better distributed fake data than GAN-BERT’s fake data distribution, as shown in Figure 1. It shows more diverse distributions for every fake data generator. Even though there are distances from the fake data, we can observe that each fake data embedding distribution shows similar distribution to the real data distribution. Moreover, each of the generators does not make matched real data distribution, and this shows that just utilizing one of them would make the classification performance poor. Additionally, as we observed in data embeddings using only the last layer of BERT, we found that only using one layer output has a limitation for learning real data distribution, Figure 7.

Furthermore, in Figure 6c,f,i,l we did not consider the k+1 class to show how the discriminator predicts an adequate label from {1,…,k} for the fake data. The visualization results show that most fake data have similar classes to the ground truths and diverse classes compared to GAN-BERT, as shown in Figure 1.

## 5. Conclusions and Future Work

This paper introduces a GAN-based approach for semi-supervised learning for text classification. LMGAN outperforms most of the GAN-based baseline performances over various text classifications by up to about 20.0%. LMGAN is a new approach to semi-supervised text classification that gives hints to the generator directly with a learned BERT. Moreover, it has multiple generators that carry different linguistic information in sentences. With this architecture, we could solve the limitation that learning with frequently changing distributions makes it difficult for the generator to make similar fake data distribution to the real data distribution. To validate our model, we show performances on various datasets. Moreover, with t-SNE visualization, we propose that LMGAN fake data embeddings show a more diverse distribution than the baseline. Additionally, we show performances of various hidden layer sets to prove that using multiple generators is meaningful and shows up to about 1.8% improvements.

However, there are remaining issues with LMGAN that could be interesting topics for future work. First, the {6,7,9} layer combination does not always show the best performances. For example, combination set {1,2,3} showed the best performances in the SST-5 dataset even if set {1,2} and set {1,2,3,4} showed poor performances. Hence, investigating the linguistic characteristics of those unique combinations could be further future work. Second, for the MNLI dataset, LMGAN is not useful for all text classification. LMGAN showed poor improvements when there are large amounts of annotated MNLI data. It only has significant performance improvements at single-sentence classification. It is desirable to investigate which hidden layers are sensitive to relationships between sentences.

## Figures and Tables

**Table 1:** Accuracy (%) of BERT for text classification with a different percentage of labeled data. All data are from the QC coarse-grained dataset.

Percentage of Labeled (%)	Accuracy (%)
1	35.3
10	73.3
50	78.8

**Table 2:** Statistics of datasets for experimentations. “#” in the category header means the amount of the corresponding data. This table contains the number of train data, test data, classes, and descriptions for each dataset.

Dataset	#Train	#Test	#Class
20 News Group	11,314	7531	20
QC coarse-grained	5500	500	6
QC fine-grained	5500	500	50
SST-5	8544	2210	5
MNLI mismatched	392,702	10,000	3
MNLI matched	392,702	10,000	3

**Table 3:** Accuracy (%) on the QC coarse-grained dataset with different amounts of annotated data with different layer sets for the generators. Results are averaged with three random seeds. The best results are in bold.

	Annotated (%)							
**Layer Set**	**1**	**2**	**5**	**10**	**20**	**30**	**40**	**50**
{12}	68.8	73.9	91.7	93.3	95.1	**96.0**	95.7	96.3
{1,2}	65.7	**75.7**	91.3	92.7	94.9	95.9	95.5	95.8
{1,2,3}	66.1	75.1	91.7	93.3	**95.9**	95.1	**96.1**	**96.7**
{1,2,3,4}	66.9	73.7	**92.7**	92.7	94.6	95.2	95.5	95.5
{9,12}	64.5	72.9	90.6	93.1	95.2	94.9	95.8	**96.7**
{6,9,12}	67.5	71.1	**92.7**	**93.4**	94.7	**96.0**	95.4	96.2
{6,8,9,12}	**69.1**	72.5	92.5	**93.4**	94.5	95.7	95.6	95.7
{6,9}	65.2	74.9	92.4	93.2	94.7	95.3	95.9	96.3
{6,7,9}	**69.1**	74.7	92.4	93.3	95.5	95.3	95.9	96.5

**Table 4:** Accuracy (%) on the SST-5 dataset with different amounts of annotated data with different layers set for the generators. Results are averaged with three random seeds. The best results are in bold.

	Annotated (%)							
**Layer Set**	**1**	**2**	**5**	**10**	**20**	**30**	**40**	**50**
{12}	34.6	40.7	46.1	51.6	**57.4**	**60.6**	61.8	62.9
{1,2}	36.5	40.6	42.6	51.0	56.5	59.4	61.0	62.4
{1,2,3}	**36.8**	41.7	45.6	51.6	57.2	60.1	**62.5**	**62.9**
{1,2,3,4}	35.4	40.6	**46.3**	49.7	56.4	60.2	61.4	62.1
{9,12}	35.5	40.9	45.1	51.6	56.9	59.0	61.8	61.9
{6,9,12}	36.4	**41.9**	45.3	**51.9**	57.3	**60.6**	61.8	62.7
{6,8,9,12}	34.6	41.5	45.8	51.6	57.0	**60.6**	61.9	61.5
{6,9}	36.0	40.8	45.1	51.6	57.1	59.5	61.2	62.0
{6,7,9}	35.9	41.8	45.6	51.6	57.3	59.8	62.2	62.7

**Table 5:** Ablation study on QC coarse-grained 1% annotated data. Performances are evaluated with three different seeds.

Model	Accuracy (%)
LMGAN	69.1
- Trained BERT of GAN-BERT at Generators	67.9
- Trained Discriminator of GAN-BERT	62.9
- Trained BERT and Discriminator of GAN-BERT	64.6
- Multiple Generators	68.8
- Cross Entropy Loss at Generator	65.0
- KL Divergence Loss at Generator	67.5
- Both Loss at Generator	61.2

**Figure A1:**
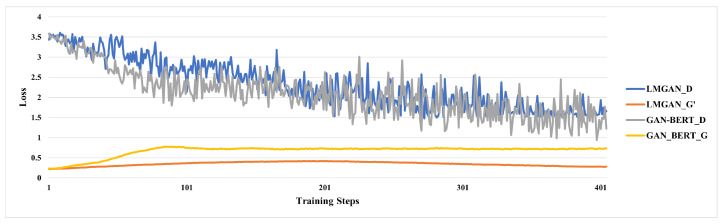
Loss plots of LMGAN and GAN-BERT. LMGAN_D and GAN-BERT_D represent values of Equation (4). LMGAN_G’ and GAN-BERT_G show values of Equation (7). In the case of GAN-BERT, the input of the generator is random noise.

## Data Availability

Publicly available datasets were analyzed in this study. 20 News Group, QC, SST-5, and MNLI data can be found here (accessed on 23 August 2022): http://qwone.com/~jason/20Newsgroups/, https://cogcomp.seas.upenn.edu/Data/QA/QC/, https://nlp.stanford.edu/sentiment/, and https://cims.nyu.edu/~sbowman/multinli/.
